# Could Sensory Mechanisms Be a Core Factor That Underlies Freezing of Gait in Parkinson’s Disease?

**DOI:** 10.1371/journal.pone.0062602

**Published:** 2013-05-08

**Authors:** Kaylena A. Ehgoetz Martens, Frederico Pieruccini-Faria, Quincy J. Almeida

**Affiliations:** 1 Department of Psychology, University of Waterloo, Waterloo, Ontario, Canada; 2 Sun Life Financial Movement Disorders Research and Rehabilitation Centre, Wilfrid Laurier University, Waterloo, Ontario, Canada; McMaster University, Canada

## Abstract

The main objective of this study was to determine how manipulating the amount of sensory information available about the body and surrounding environment influenced freezing of gait (FOG), while walking through a doorway. It was hypothesized that the more limited the sensory information, the greater the occurrence of freezing of gait. Nineteen patients with Parkinsoǹs disease who experience freezing of gait (PD-FOG) walked through a doorway or into open space in complete darkness. The three doorway conditions included: (i) FRAME (DARK) – walking through the remembered door frame; (ii) FRAME - walking through the door with the door frame illuminated; (iii) FRAME+BODY - walking through the door (both the door and the limbs illuminated). Additionally, two conditions of walking away from the doorway included: (iv) NO FRAME (DARK) - walking into open space; (v) NO FRAME+BODY - walking into open space with the limbs illuminated, to evaluate whether perception (or fear) of the doorway might account for FOG behaviour. Key outcome measures included: the number of freezing of gait episodes recorded, total duration of freezing of gait, and the percentage of time spent frozen. Significantly more freezing of gait episodes occurred when participants walked toward the doorway in complete darkness compared to walking into open space (p<0.05). Similar to previous studies, velocity (p<0.001) and step length (p<0.0001) significantly decreased when walking through the door in complete darkness, compared to all other conditions. Significant increases in step width variability were also identified but only when walking into open space (p<0.005). These results support the notion that sensory deficits may have a profound impact on freezing of gait that need to be carefully considered.

## Introduction

Animal models of Parkinson’s disease (PD) have supported the notion that sensory processing may be related to the degree of striatal dopamine loss [1], [2]. Keijsers and colleagues (2005) tested a spectrum of human PD patients with a pointing task in complete darkness, comparing pointing with an illuminated frame of reference to pointing with an LED on the fingertip (providing additional visual feedback). This study showed that patients with PD performed poorly (compared to healthy control participants) regardless of condition, suggesting that PD patients may have a limited ability to utilize proprioceptive information to reduce movement variability [3].

Gait variability has been identified as an important parameter linked to freezing of gait (FOG), a severe symptom affecting over 50% of patients with advanced Parkinson’s disease (PD) [4], [5]. Interestingly, in comparison to healthy age-matched control participants (and PD patients that do not experience FOG), PD-FOG have been shown to be profoundly influenced by doorways. This has been indicated by drastic reductions in velocity, step length as well as increases to step time, and more importantly, a multitude of gait variability measures that may be indicative of an upcoming FOG episode [6]–[8]. Thus it is important to consider whether PD patients experiencing FOG might have difficulty utilizing sensory feedback, given that some of the most common situations where FOG occurs involves a change in the visual environment, and hence might require integration of visual and proprioceptive information (e.g. turning, passing through narrow spaces such as a doorway) [5], [9], [10].

It remains unclear why FOG occurs, although a variety of theories have been proposed such as experiencing a motor block [9], failure to shift motor programs [7], [11], problems with bilateral coordination [12], deficits in visuomotor processing [13], executive and attentional dysfunction [14]–[16]. It may be important, however, to consider the commonalities that may exist between these explanations. In all of these theories, sensory deficits could potentially be associated with an underlying mechanism for FOG [6], [13], [17]. For example, the bilateral coordination hypothesis focuses on describing an observation of behaviour rather than considering how a sensory feedback deficit (causing a PD patient to be less aware of step timing discrepancies between the legs) might propagate FOG. In addition, it may also be important to consider whether the sensory systems may be necessary in evaluating perceived threats from obstacles and objects that we need to avoid in the environment. Given that FOG is anecdotally reported to occur in crowded and confined spaces (e.g. doorways and elevators) this perspective may be critical to consider. Thus, it is essential to explore how specific sources of sensory feedback derived both from body and the external environment might contribute to FOG.

The main objective of this study was to evaluate how the availability of sensory feedback from different sources might contribute to locomotion and FOG episodes in PD. Some researchers have suggested that severe PD adapt to operate in a proprioceptive frame of reference during movement, but perform poorly because of sensory processing deficits [3]. It was hypothesized that if proprioception contributes to FOG, then FOG should occur most frequently during conditions where individuals must walk in complete darkness, since during these conditions, participants are forced to rely primarily on proprioception rather than vision [18]. Illuminating both the doorframe and participants’ limbs, will provide participants with visual information about the relation of self to an external frame of reference [3]. This manipulation will allow us to evaluate whether PD-FOG will improve their gait and experience less FOG episodes since the additional visual information might override the need to rely on other faulty sensory information. Since the current study was focused on factors that contribute to FOG, we investigated patients experiencing FOG while walking toward a doorway (a potentially threatening obstacle in the environment) and into open space, while varying the amount of visual information available.

## Materials and Methods

### Patients

This study involved 21 patients with PD, who were confirmed to experience FOG using the following criteria: (i) previous diagnosis of idiopathic PD by a neurologist and history of FOG; (ii) patients self-reported FOG using UPDRS-II; (iii) a movement disorder specialist confirmed the presence of FOG during patient assessment (prior to participation in the study see [15] for procedure). All patients were recruited from the patient database at the Sun Life Financial Movement Disorders Research and Rehabilitation Centre at Wilfrid Laurier University in Waterloo, Canada. Participants were excluded from the study if they had visual disturbances that would impair their ability to see a doorway, or if they were unable to walk unassisted for 7 m. Patient files were also carefully screened for co-morbid conditions (i.e. history of stroke, visual impairments, hearing loss, cognitive decline, peripheral neuropathies, diabetes and dementia) prior to participation. Since there has not been conclusive evidence showing that FOG is responsive to dopaminergic medication, and our primary research questions was to understand how sensory feedback influenced FOG, it was most ecologically valid to test participants in the ON state since this is typically their medication state during their daily activities. For these reasons, all participants were tested 1 hour after taking their regular dosage of anti-Parkinsonian medication, with the exception of one patient who was not being treated for PD at the time (see Table 1).

**Table 1:** Parkinson’s disease participant characteristics including age, sex, clinical evaluation using the motor section of the Unified Parkinson’s Disease Rating Scale, Modified Mini Mental State Exam, Corsi Working Memory Tapping Test, current Parkinson medication, and the total number of freezing of gait episodes experienced.

Participant	Age	Sex	UPDRS-III	3MS	Corsi WMT	Medications	Dosage (mg)	FOGExperienced	Total#FOG
**1**	62	M	41	93	2	Sinemet	312.5	Y	20
**2**	65	F	31	–	–	Sinemet	125	Y	1
**3**	70	M	34	87	4	Sinemet	125	N	0
**4**	83	M	38	89	4	Sinemet	187.5	N	0
**5**	80	M	33	76	3	Sinemet	375	Y	8
**6**	78	M	27	86	4	Sinemet	312.5	Y	1
**7**	72	M	23	89	3	mirapex	125	Y	2
**8**	71	M	20	91	4	Sinemet	187.5	N	0
**9**	75	M	34	78	2	no meds	0	N	0
**10**	83	M	40	88	3	Sinemet	375	Y	10
**11**	77	M	35	–	–	Sinemet	125	N	0
**12**	59	F	25	–	–	sin/com	187.5/200	Y	2
**13**	73	M	29	98	4	sin/com	250/100	N	0
**14**	68	M	36	–	–	Sinemet	312.5	Y	1
**15**	82	F	40	–	–	sin/com	312.5/200	Y	32
**16**	78	M	49	98	6	sinemet	125	N	0
**17**	78	F	51	–	–	sin/mir	125	Y	37
**18**	79	M	32	–	–	Stalevo	125	N	0
**19**	78	M	36	96	5	sin/trihex	187.5	Y	39
**Average**	74	15M	34	89.1	3.7		195.3	11 Y	7.7

Two participants were excluded from data analysis. One patient was unable to complete the experiment and requested to drop-out since she eventually became completely frozen in the dark and was unable to walk until the lights were turned on. The second patient was excluded from analysis since his diagnosis of PD was being reconsidered during the time of testing. One week following his diagnoses with PD had been withdrawn. The data from these two participants was discarded, and thus a total of 19 participants were included in the data analysis. All participants were informed about the experimental protocol and written consent was obtained prior to participating in this study according to the Declaration of Helsinki. This study received full ethical approval by the Research Ethics Board at Wilfrid Laurier University.

### Experimental Setup

Participants stood in a large gymnasium in complete darkness in two pathway conditions (both 7 m in length): (i) FRAME – walking toward a standard doorway (91 cm) which entered a confined space, (ii) NO FRAME – the identical 7 m path but starting at the doorway and walking into a large open space. To our knowledge, this is the very first study to synchronize six OPTOTRAK® (Northern Digital, NDI, Waterloo, ON) cameras around the blackened room to capture full body kinematics at a frequency of 100 Hz. IRED markers were placed in clusters (a 3-marker rigid body) on the xiphoid process, T7 vertebrae, anterior tibias, gastrocnemius, and individual markers were placed on the heels, 1st and 5th metatarsals. The doorway in the environment was bordered with rope lighting (3/8″ heavy-duty PVC extruded core flexible rope light) controlled with an intensity switch, which was set at a dim illumination. This restricted the amount of light available to only the door frame, but not the surrounding dark environment. Glow in the dark Velcro® strips were also used and attached to participants forearms (with the use of a Velcro® sleeve), thighs, and feet during certain experimental conditions. Between every trial, a very bright light was turned on and pointed at participants, not only to ensure a safe return to the appropriate start position, but this also prevented participants’ eyes from adapting to the darkness. Dark adaptation could not have occurred since the lights did not stay off for more than one trial at a time (on average each trial took approximately 15 seconds), and was always followed by shining a bright projector light toward their face.

### Experimental Paradigm

**Figure 1:**
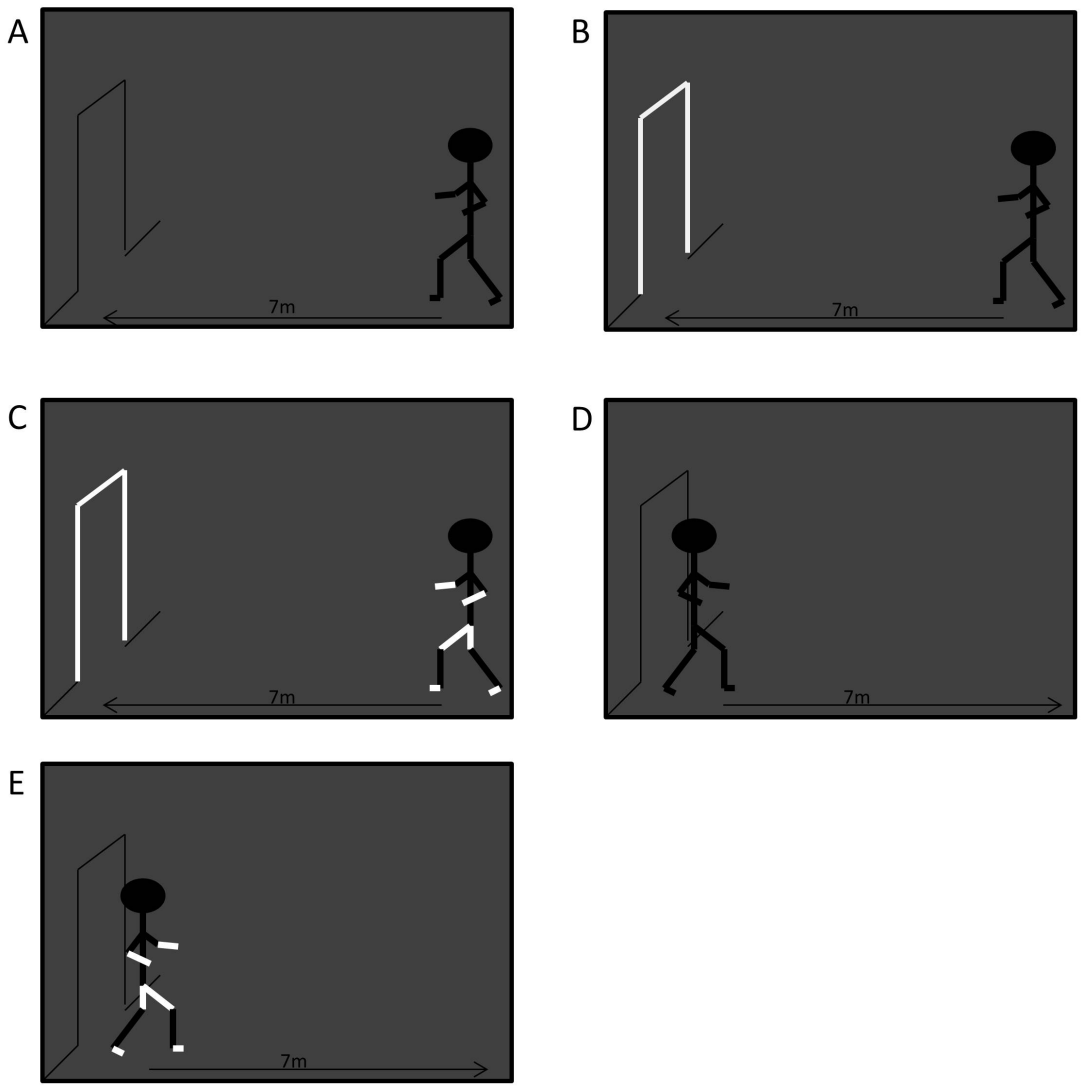
Experimental Conditions. A) participants walk in complete darkness toward doorframe-FRAME (DARK); B) participants walk in complete darkness toward lit doorframe- FRAME; C) participants walk in complete darkness with limbs and doorframe illuminated- FRAME+BODY; D) participants walk in complete darkness away from the doorframe into open space- NO FRAME (DARK); E) participants walk in complete darkness with limbs illuminated away from the doorframe into open space- NO FRAME+BODY.

The experimental paradigm is a combination of the procedures of Keijsers et al., (2005) and Almeida et al., (2005). Participants completed 15 randomized walking trials in 5 different conditions of complete darkness (See Figure 1). While walking 7 m toward the doorway (91 cm wide), conditions were further broken down into a progressive spectrum of sensory feedback: (i) FRAME (DARK) – walking through the remembered door frame in complete darkness; (ii) FRAME - walking through the door with only the door frame illuminated; (iii) FRAME+BODY - walking through the door, with both the door and the limbs of the body illuminated.

Since the above mentioned trials involved walking toward a door frame in darkness, an additional set of conditions were included to evaluate whether perception (or fear) of the doorway might account for FOG behaviour. Thus, two conditions of walking 7 m away from the doorway were possible: (iv) NO FRAME (DARK) - walking into open space in complete darkness; (v) NO FRAME+BODY - walking into open space with the limbs of their body illuminated.

In all cases, participants were instructed to walk either through the doorway, or into the open space (until the experimenter told them to stop) in which case the lights were turned on and the participant returned to the start position. A spotter followed participants to ensure participants safety. Since the environment was completely dark the spotter had to use the small green light from the wireless transmitter on the back of the participant to monitor the participant during spotting. This light was not visible to participants.

### Data Analysis

Five conditions (3 FRAME, 2 NO FRAME) were compared to evaluate if their effects on FOG occurrence could be accounted for by sensory feedback manipulations alone, or if threat of the approaching doorway itself might also contribute. The primary outcome measure was the frequency of FOG which was measured both objectively and subjectively similar to Cowie et. al. (2012) [8]. A FOG episode was defined as any period where the gait velocity (measured from the IRED on the xiphoid process) dropped between a zero velocity (i.e. completely stopped) and one standard deviation of their regular velocity above zero. This criterion was devised to be a stringent objective measure of FOG however without missing episodes where the body continues to oscillate while the feet are “glued” to the floor. This objective measure was followed up use subjective confirmation by reviewing the OPTOTRAK playback of each trial [19]. Two separate raters independently visually identified freezing episodes through video observation, and then subsequently verified the number of freezing episodes between raters for complete agreement. In the event of any disagreement between raters, the episode was not considered a freeze in the current data set, thus providing 100% confirmation of all FOG episodes. These procedures allowed us to quantify the number of freezing of gait episodes in each condition, in addition to the total time and percentage of time spent frozen. These variables were analyzed using MATLAB 7.0. Initiation freezing and freezing after the door were not included in this count.

Previous research has found that spatial and temporal aspects of gait can be indicative of an upcoming FOG occurrence [6], [20]. These changes have been well established to occur only in FOG patients but not healthy age-matched controls or PD patients that do not experience FOG [6]–[8]. Furthermore since freezing of gait is difficult to evoke in an experimental setting it is also important to understand changes in gait behaviour that may not result in a full-blown freezing episode in response to the experimental manipulations. For these reasons, we chose to also analyse participants’ gait characteristics for comparison between conditions. It should be noted that during all FRAME conditions, gait was only analysed prior to crossing the doorframe. The dependent gait variables analyzed were gait velocity (cm/s), mean step length (cm), step length variability (within trial step-to-step standard deviation), mean step width (cm), step width variability (CV), step time (s), step time variability (CV). Heel strikes and toe offs were identified with a protocol defined by O’Connor, Thorpe, O’Malley, & Vaughan, 2007 [21]. Gait variables were analyzed using repeated measures ANOVA with 2 factors of repeated measures (5 sensory conditions ×3 trials).

The frequency of FOG episodes, the total duration of time spent frozen, and the percent of trial spent frozen were analyzed using repeated measures ANOVA with 1 factor of repeated measures (allowing a comparison of the FOG variables between the five conditions). In order to compare the frequency of FOG occurrences, the total number of FOG episodes was counted across all trials within each condition and compared within-subject. Likewise, the duration and percent of trial spent frozen were averaged across trials within each condition and compared within-subject. In all cases, Tukey’s HSD post hoc procedure was used to further investigate significant differences.

## Results

Demographic data of our FOG population are summarized in Table 1. FOG episodes were experienced by in 53% of all participants for a total of 151 episodes.

### Freezing of Gait Variables

**Figure 2:**
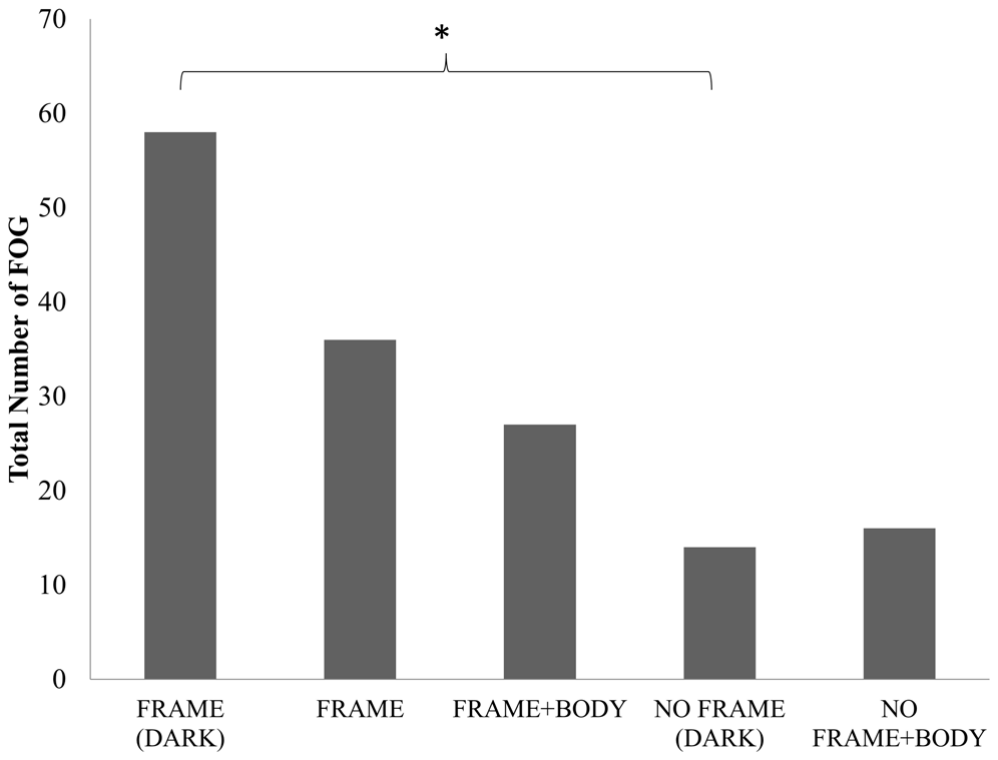
Total Number of FOG episodes. Illustrates total number of freezing of gait episodes that occurred during locomotion toward the doorway or into open space with different amounts of sensory feedback. *represents significant difference at the p<0.05 level.

Figure 2 demonstrates the dynamic effect of sensory feedback on the occurrence of FOG across conditions. A main effect of condition was found for the frequency of FOG occurrences (F(4,72) = 16.15, p = 0.047). Post hoc analysis revealed that the only conditions that were significantly different from each other were the FRAME (DARK) condition compared to the NO FRAME (DARK) condition (p = 0.046). It should be noted that in some trials of walking toward the door, FOG episodes were so severe that the trial could not be completed and thus it was necessary to repeat the trial (note that there were no occurrences while walking away from the doorway into open space). No significant differences were found when comparing the total duration of FOG (F(4,72) = 3.72, p = 0.84) or the % of trial spent frozen (F(4, 72) = 37.78, p = 0.17) across conditions. However, given that percentage of time spent frozen has been deemed to be a more reliable measure [22], it is important to note that a planned comparisons analysis of the percentage of time spent frozen between FRAME (DARK) and NO FRAME (DARK) conditions, also confirms a significant difference in the amount of FOG (t(18) = 1.73, p = 0.016).

### Gait Variables

#### Velocity

A main effect of condition was identified (F(4,72) = 5.89, p<0.001), and post hoc analysis confirmed that patients walked significantly slower when walking toward the door in complete darkness (FRAME DARK) compared to all other conditions. A main effect of trial was also identified (F(2,36) = 16.25, P<0.0001), and post hoc analysis confirmed that patients significantly increased their speed as the trials progressed.

#### Step length

As expected, there was a significant difference in mean step length when comparing the five conditions (F(4,72) = 7.86, P<0.0001). Post hoc analysis confirmed that patients significantly shortened their step length when walking toward the door in complete darkness (FRAME DARK) compared to all other conditions. A main effect of trial was also identified (F(2,36) = 11.21, P<0.001), with post hoc analysis revealing that patients significantly shortened their step length during the first experience with a condition. There were no statistically significant differences when comparing the mean step length variability.

#### Step width

A main effect of condition was found with regards to step width (F(4,72) = 5.37, P<0.001). Step width decreased in both conditions where the door was illuminated (FRAME and FRAME+BODY) when compared to walking into open space with the body lit (NO FRAME+BODY).

**Figure 3:**
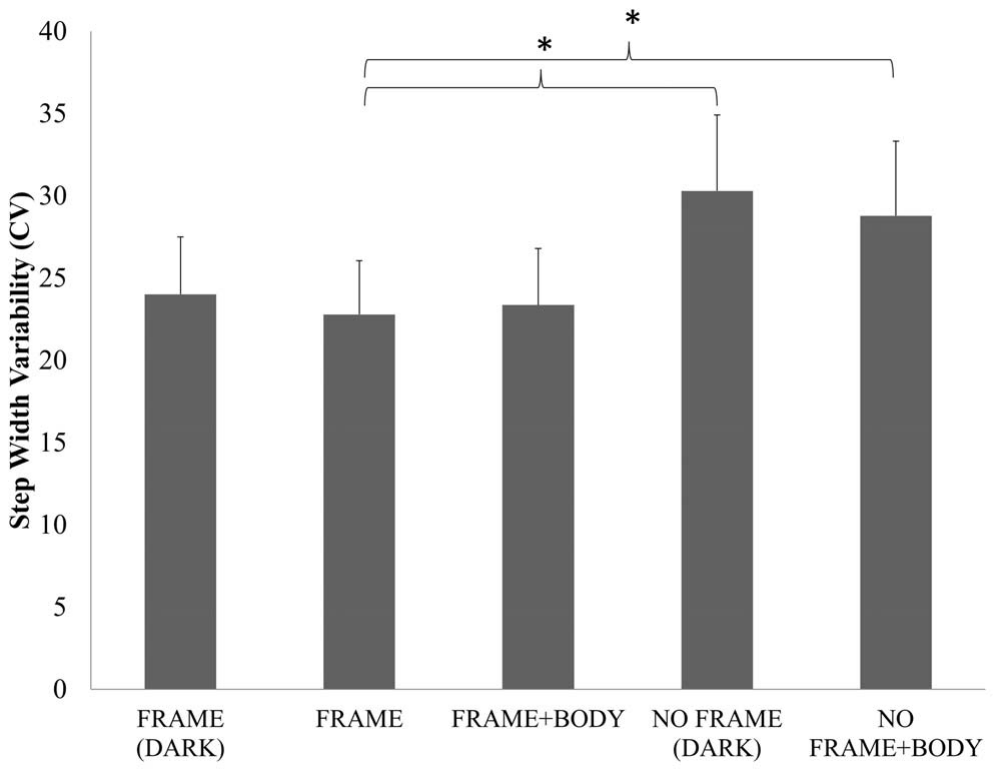
Step Width Variability. Illustrates the average step width variability during locomotion toward the doorway or into open space with different amounts of sensory feedback. Error bars represent standard error. *represents significant difference at the p<0.05 level.

A main effect of condition for step width variability was also found (F(4,72) = 4.14, P<0.005). Figure 3 demonstrates that patients displayed increased variability when walking into open space either with their body illuminated (NO FRAME+BODY) and in complete darkness (NO FRAME DARK) compared to when patients walked toward the lit door (FRAME), which was confirmed using Tukey’s post hoc analysis (See Figure 3).

#### Step time

While there were no significant results for step time, a main effect of condition for step time variability was nearly significant (F(4,72) = 2.43, P = 0.056). Similar to the results for FOG occurrences, patients displayed increased step time variability when walking toward the door in the complete darkness (FRAME DARK) compared to patients walking into open space with visual information about their body (NO FRAME+BODY).

## Discussion

To our knowledge, this is the first study to investigate how darkness might impact FOG behaviours in PD, and over 150 freezing episodes were captured utilizing this protocol. The main purpose of this experiment was to evaluate how availability of sensory information from the surrounding environment (frame of reference) and self (body) might influence FOG and characteristics of gait just prior to an FOG episode. It was hypothesized that without vision, a greater number of FOG episodes might occur since PD patients would be unable to build an appropriate model of self-motion without a visual frame of reference (whether body or frame-centered) [3]. In support of this hypothesis, the greatest number of FOG episodes occurred while approaching the door frame in complete darkness, furthermore FOG occurrences were reduced by 38% with an external frame of reference available (i.e. visual outline of the door frame). FOG occurrences were further reduced by an additional 25% when limb position was illuminated, thus allowing vision to enhance the existing proprioceptive signal. Some alternative theories have argued that PD-FOG exaggerate their responses to visual information, resulting in a FOG episode [13], however the current results provide an interesting contrast suggesting that visual information might help reduce FOG (since illuminating the door reduced FOG by 38% compared in walking through the door in complete darkness). Although these improvements were not significant, they may be clinically relevant and may provide further insight into the mechanism of FOG.

Also in support of our hypothesis, changes were identified in gait parameters (i.e. velocity, step length) prior to passing through the doorway, specifically, both velocity and step length were significantly reduced while walking toward the doorway in complete darkness, in contrast to all other conditions where a visual reference of the limbs or a door frame were available. These findings are consistent with previous research that has investigated gait changes while walking toward a doorframe specifically in participants with PD that experience FOG [6], [8], [13]. This supports the notion that vision allows for an opportunity for proprioceptive feedback to become more relevant during gait.

However, an interesting alternative hypothesis may be that patients who suffer from FOG are most affected when a significant threat exists (but sensory information available is not sufficient to assure safety). This is in accordance with the perspective of Wolpert and colleagues [23]–[25] describing the formulation of an internal model. In order to evaluate this hypothesis, we compared walking in complete darkness while walking toward the doorway (FRAME DARK) to walking away from the doorway (NO FRAME DARK). A significant difference in FOG occurrences between these two visually identical conditions revealed that patients froze four times more often when walking toward the door frame, than walking into open space (yet both of these conditions were in complete darkness). Additionally, step time variability, which has been previously linked to cognitive load processing during FOG [20], was also increased while walking toward the doorway (compared to both conditions when walking away from doorway). This finding is further reinforced by the fact that step timing was the least variable while walking away from the doorway with the addition of limb illumination. Interestingly, an increase in step width variability was identified in both conditions where participants walked into open space (i.e. NO FRAME) relative to the condition with only a view of the external frame of the doorway being outlined (i.e. FRAME). Since visual information is typically used to make adjustments to step parameters [26], [27] it might have been expected that step width variability would be higher when walking toward the door, since visual feedback was available for online correction. However, Keijsers et al. might argue that when there is no visual or remembered frame of reference, severe PD patients are forced to operate with a primarily proprioceptive frame of reference [3]. Hence, increases to step width variability might be expected during conditions where participants are walking into open space, since no environmental frame of reference is available. These findings further exemplify the importance of sensory feedback during navigation without vision in PD-FOG. Thus, it is important to consider why, even in darkness, the knowledge of an upcoming doorway might lead to FOG behaviour. These sorts of responses, although potentially driven by fear, are likely related to an inability to link perception to action during movement in PD-FOG. According to this perspective, one might expect that perception in itself is impaired in PD. However, recent research has demonstrated that perception is only impaired when proprioception is a primary source of sensory information driving the eventual movement response [28]. It is also important to consider how an internal model may need to be created to determine how movement is to be executed with or without the presence of a doorway. If in both of these conditions a feedforward model was created prior to the initiation of movement, then we would not expect differences in performance between these two conditions. However, since the number of freezes significantly increases while walking toward the doorway, it seems likely that PD-FOG patients must be attempting to make use of proprioceptive feedback to guide to performance expected from their internally derived model. Future research may need to carefully consider how cognitive, affective and perceptual mechanisms might trigger FOG, especially when walking in complete darkness.

Since the greatest number of FOG episodes and step time variability was seen when walking toward the door in complete darkness, it is possible that FOG-patients were experiencing greater cognitive load in order to monitor and integrate proprioceptive information (in the absence of vision) with knowledge of the doorway, similar to a dual task. Participants must capture coordinates of the door and integrate this information into the motor plan while continually updating body location in order to adjust proceeding steps. This involves increased sensory processing and integration to successfully monitor movements toward the door. Evidence from deafferented patients showed that a lack of proprioceptive information can increase cognitive effort to generate steps cycles [29]. Patients with PD also have more difficulty walking to a remembered target in complete darkness when they had to rely primarily on proprioceptive feedback to navigate to the target [18]. A recent study demonstrated reduced cerebral activity in the right superior parietal lobule in PD during imagery of gait [30], lending support to the hypothesis that sensorimotor integration may be impaired in patients with FOG when there is no frame of reference [31]. However, since all the patients knew whether they would be walking toward a door or into open space at the onset of any given trial, an alternative explanation might be that participants felt more threat when walking in darkness toward a potential obstacle. From this perspective, it is important to note that FOG episodes did occur even while walking in darkness toward an open space, suggesting that fear of a potential collision might lead to a more cautious gait that attempts to collect as much sensory feedback as possible during locomotion. In the case of knowing that walking toward a door in complete darkness, the fear of a potential collision must be enhanced and thus explaining the significant increase in FOG episodes. Even from this fear perspective it becomes clear that individuals with PD must be attempting to utilize sensory feedback in order to optimize safety when avoiding hazards in their environment. Both of these perspectives are important to consider in order to fully understand how previous theories of FOG behaviours might be the result of a common sensory mechanism. Taken together, the main conclusion of this study is that sensory processing deficits may lie at the very core of many of the proposed theories of freezing of gait.
